# Compensation claims after knee cartilage surgery is rare. A registry-based study from Scandinavia from 2010 to 2015

**DOI:** 10.1186/s12891-020-03311-4

**Published:** 2020-05-08

**Authors:** Tommy Frøseth Aae, Øystein Bjerkestrand Lian, Asbjørn Årøen, Lars Engebretsen, Per-Henrik Randsborg

**Affiliations:** 1 Department of Orthopedic Surgery, Kristiansund Hospital, 6518 Kristiansund, Norway; 2 Institute of Clinical Medicine, Faculty of Medicine, University of Oslo, Oslo, Norway; 3 Institute of Neuromedicine, Faculty of Medicine, Norwegian University of Science and Technology, 7491 Trondheim, Norway; 4 Department of Orthopedic Surgery, Akershus University Hospital, 1478 Lørenskog, Norway; 5 Institute of Clinical Medicine, Campus Ahus, University of Oslo, 1478 Lørenskog, Norway; 6 Oslo Sports Trauma Research Center (OSTRC), Norwegian School of Sports Sciences, postbox 4014 Ullevål Stadion, 0806 Oslo, Norway; 7 Department of Orthopedic Surgery, Oslo University Hospital, 0450 Oslo, Norway

**Keywords:** Articular cartilage, Microfracture, Autologous chondrocyte implantation, Compensation claim

## Abstract

**Background:**

Focal cartilage defects (FCDs) in the knee joint has a high prevalence. A broad range of treatment options exists for symptomatic patients. Knowledge of patient compensation claims following surgical treatment of FCDs is missing. The purpose of this study is to evaluate compensation claims filed to the Scandinavian registries for patient compensation following treatment of FCDs in the knee joint from 2010 to 2015 and identify possible areas of improvement.

**Methods:**

A cross-sectional study design was used to obtain all complaints following surgical treatment of FCDs from the Scandinavian registries from 2010 to 2015. Data such as age, gender, type of treatment, type of complaint, reason of verdict and amount of compensation were collected and systematically analyzed.

**Results:**

103 patients filed a compensation claim. 43 had received debridement (41.7%), 54 microfracture (MF) (52.4%), 3 mosaicplasty (2.9%) and 3 autologous chondrocyte implantation (ACI) (2.9%). Of the 103 claims, 36 were granted (35%). 21 following debridement (58.3%), 13 after MF (36.1%), 1 following mosaicplasty (2.8%) and 1 after ACI (2.8%). The most common reason for complaint was infection (22.1%), of which 89% were granted. The average compensation was €24.457 (range €209 – €458.943).

**Conclusion:**

Compensation claims following surgical treatment of knee cartilage injuries in Scandinavia are rare. Establishing nationwide cartilage registries can add further knowledge on this troublesome disease.

## Background

Focal cartilage defects (FCDs) in the knee joint is a high prevalence injury that may cause pain and reduced function, with the risk of early onset osteoarthritis [1–3]. Various surgical treatment options are available. The goal of operative treatment is to restore the articular cartilage and reduce symptoms and minimizing the risk of osteoarthritis [4, 5]. Surgical treatment relieves symptoms, but regardless of surgical procedure, the majority of patients do not achieve normal knee function [6–8]. No method or treatment has proved to be superior to any other, and there is currently no gold standard or consensus on what constitutes the best treatment for FCDs of the knee [9–11].

Orthopedic surgery is one of the medical specialties with the highest rate of compensation claims following medical treatment [12]. Consequently, there is an increased interest in compensation claims related to orthopedic surgery [13, 14]. Previous studies have mainly reported compensation claims following hip and knee arthroplasty and spine disorders [15, 16]. One study has reported malpractice litigation following arthroscopic surgery in general, but to the best of our knowledge, no study has previously reported compensation claims following FCDs in the knee specifically [17].

The purpose of this study is to evaluate compensation claims filed to the Scandinavian registries following surgical treatment of FCDs in the knee joint from 2010 to 2015 and identify possible areas of improvement. We hypothesized that compensation claims are more frequent after debridement and microfracture (MF) compared to mosaicplasty and autologous chondrocyte implantation (ACI).

## Methods

### Data source

In Scandinavia, compensation claims for injuries in connection with medical treatment are handled by nationwide systems. The compensation principle in these nations is a no-blame system based on the principle of avoidability (i.e. if the injury sustained during treatment was avoidable). A no-blame compensation principle separates the compensation issue from legal malpractice, permitting most indemnity cases in Scandinavia to be settled outside the judicial system. In Norway, the complaints are handled by the Norwegian System of Patient Injury Compensation (NPE) [18]. Patients can appeal against a decision to the Patient Injury Compensation Board, which is under the Ministry of Health. In Sweden, the claims are processed by the National Swedish Patient Insurance Company, a mutual company owned by the counties [19]. In Denmark, the Patient Insurance Association handles claims concerning malpractice and injuries, as well as injuries caused by medical products [20].

In all three systems, compensation is only considered if three conditions are met [18]. Firstly, the injury must have been caused by the examination, diagnosis, treatment (or lack of treatment) or follow-up of the condition. The treatment (or lack thereof) must be deemed erroneous or substandard compared to current treatment guidelines. If the reason for complaint is considered to be a consequence of the primary injury, and not the treatment, compensation is not granted. There is one exception to this rule (the reasonability rule). This exception permits compensation to be granted after rare and serious complications even if no treatment failure can be identified. Secondly, the patient must have led a substantial financial loss in excess of what would otherwise be expected. Thirdly, the claim must be put forward within a reasonable time (currently set to 10 years in Sweden and three years in Denmark and Norway). These similar conditions enable us to combine data from all three Scandinavian countries in our analysis.

### Participants

**Fig. 1:**
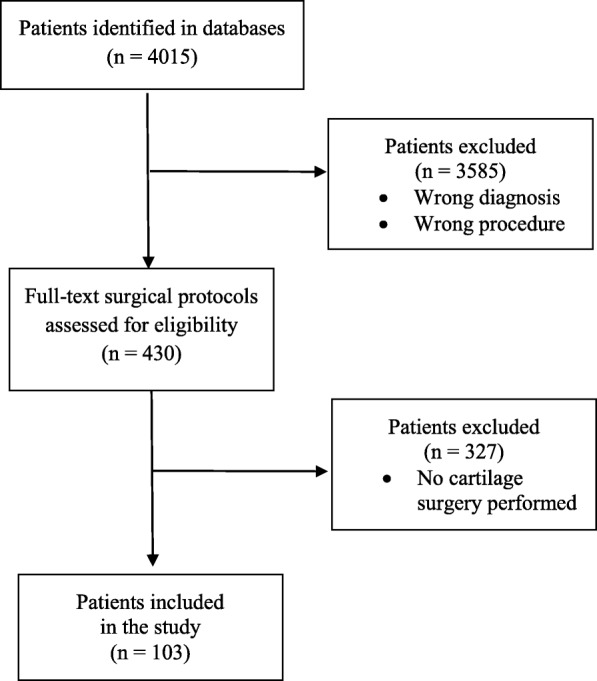
Flow diagram of patient’s selection included in the study

Data from the Danish, Norwegian and Swedish nationwide registries were obtained from each of the respective national registries. Patients of both genders and of any age who filed a compensation claim following articular cartilage surgery of the knee from 2010 to 2015 were considered for inclusion. The nationwide databases were searched for a predefined set of diagnosis and surgical procedures using the International Statistical Classification of Diseases and Related Health Problems 10th Revision (ICD-10) and the NOMESCO Classification of Surgical Procedures (NCSP) Version 1.14 [21, 22] (Table 1). The potential patient files were subsequently screened by the corresponding author, identifying patients who had been treated for an isolated cartilage defects of the knee. The surgical notes were then reviewed before final inclusion (Fig. 1).

**Table 1:** Overview of the predefined diagnosis and surgical procedures using the ICD-10 and NCSP codes

Diagnosis	Surgical procedures
M17 Gonarthrosis	NGA11 Endoscopic exploration
M22.4 Chondromalacia patella	NGA12 Open exploration
M23.4 Loose body in the knee	NGF21 Endoscopic fixation of corpus librum
M23.8 Other internal derangements of knee	NGF22 Open fixation of corpus librum
M23.9 Internal derangement of knee, unspecified	NGF31 Endoscopic resection of corpus librum
M24.1 Other articular cartilage disorder	NGF32 Open resection of corpus librum
M24.8 Other specific joint derangements, not elsewhere classified	NGF91 Other endoscopic procedure on synovia or articular cartilage
M24.9 Joint derangements, unspecified	NGF92 Other open procedure on synovia or articular cartilage
M25.5 Pain in joint	NGG29 Other arthroplasty without prosthesis
M25.8 Other specified joint disorders	NGG99 Other excision, reconstruction or arthrodesis of knee
M25.9 Joint disorder, unspecified	NGH41 Endoscopic removal of corpus librum
M92.4 Juvenile osteochondrosis, unspecified	NGH42 Open removal of corpus librum
M92.8 Other specified juvenile osteochondrosis	NGH91 Other endoscopic procedure
M92.9 Juvenile osteochondrosis, unspecified	NGH92 Other open procedure
M93.2 Osteochondritis dissecans	NGK09 Excision of bony fragment in knee
M93.8 Other specified osteochondropaties	NGK19 Resection or excision of bone in knee
M93.9 Osteochondropathy, unspecified	NGK29 Fenestration or drilling of bone in knee
S83.3 Tear of articular cartilage of knee, current	NGN09 Autotransplantation of bone to knee
	NGN49 Transplantation of cartilage, periost or fascia to knee
	NGN99 Other transplantation to knee
	YNA20 Removal of cartilage for transplantation
	ZZG00 Cartilage transplantation

The age, gender and nationality of the patients were collected, together with the type of treatment, type of complaint and the amount of compensation in granted cases. The reasons given for granted or rejected claims were reviewed and systematically analyzed.

### Statistics

Mean, median and standard deviation were calculated for continuous variables. Categorical data were presented in frequencies and cumulative frequencies. Groups were compared using the independent t-test or the Chi-square test. A *p*-value < 0.05 was considered statistically significant. The analysis was performed using IBM SPSS Statistics v25.

## Results

**Fig. 2:**
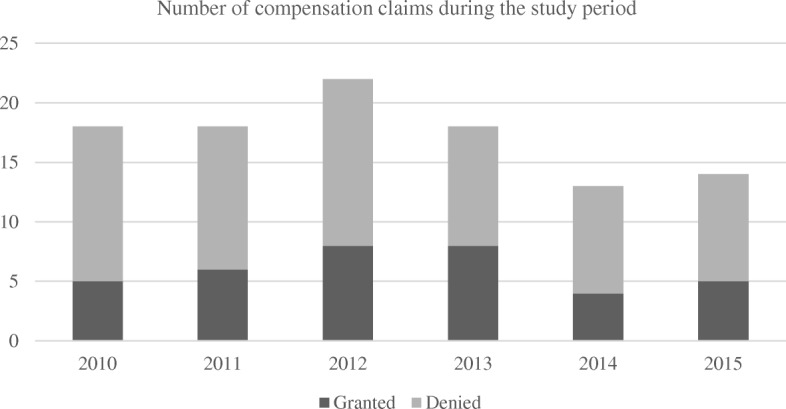
Complaints filed to the Scandinavian registries following surgical treatment of focal cartilage defects in the knee joint between 2010 and 2015

**Fig. 3:**
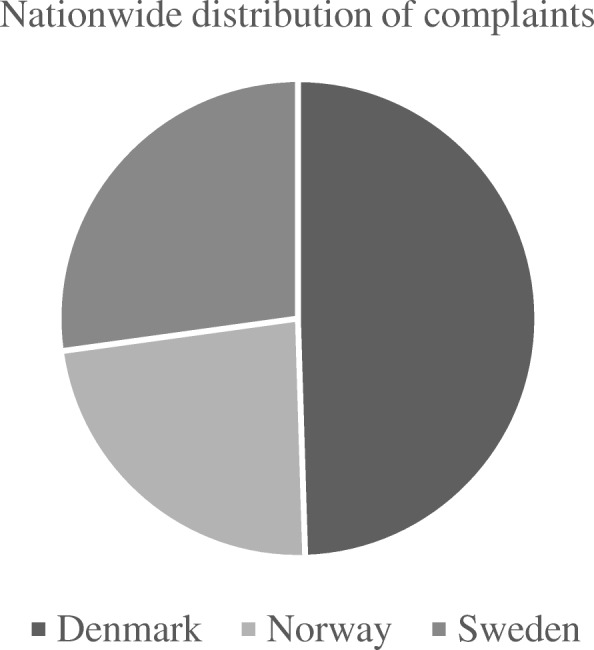
Nationwide distribution of complaints put forward to the Scandinavian registries following surgical treatment of focal cartilage defects in the knee joint between 2010 and 2015

We identified 103 compensation claims put forward to the registries following articular cartilage surgery in the knee from 2010 to 2015 (Fig. 1). There was a slight decrease in claims for compensation the last two years of the study period (Fig. 2). Most claims were put forward to the Danish registry (Fig. 3).

The average age at the time of surgery was 38.6 years (11–71). 62 (60.2%) claims were put forward by females (Table 2). Claims following debridement (43, 41.7%) and MF (54, 52.4%) was far more common than following mosaicplasty (3, 2.9%) and ACI (3, 2.9%).

**Table 2:** Age and gender partitioned by declined or rejected claims following surgical treatment of focal cartilage defects in the knee joint

	Declined, ***n*** = 67 (65%)	Granted ***n*** = 36 (35%)	
Age, mean (SD, range)	38.5 (10.7, 11–71)	38.8 (12.1, 13–55)	0.93
Females, n (%)	41 (61.1%)	21 (33.9%)	0.77

Of the 103 claims, 36 were granted (35%). There was no statistically significant difference in granted claims between males and females (15/41 versus 21/62, *p* = 0.8). 21 of the patients with granted claims were treated with debridement (58.3%), 13 with MF (36.1%), 1 with mosaicplasty (2.8%) and 1 underwent ACI (2.8%). Infection (22.2%), pain (16.7%), delayed diagnosis or treatment (13.9%), treatment failure (11.1%) and numbness (11.1%) dominated patients’ reasons for complaints (Table 3).

**Table 3:** Patients’ reasons for complaint in 36 granted claims following surgical treatment of focal cartilage defects in the knee joint

Reason for complaints (granted)	***N*** = 36 (%)	
Infection	8 (22.2%)	
Pain	6 (16.7%)	
Delayed diagnosis or treatment	5 (13.9%)	
Treatment failure	4 (11.1%)	
Numbness	4 (11.1%)	
Spinal headache	2 (5.6%)	
Stiffness	1 (2.8%)	
Swelling	1 (2.8%)	
Lack of information	1 (2.8%)	
Infected peripheral vein catheter	1 (2.8%)	
Failure of medical equipment	1 (2.8%)	
Deep vein thrombosis	1 (2.8%)	
Frozen shoulder	1 (2.8%)	

Of the patients claiming for compensation due to infection, 89% were granted, whereas for pain, only 14% of the claims were granted.

29 patients received compensation related to surgery (such as infection or inadequate surgical technique), whereas 7 patients received compensation unrelated to surgery (such as delayed diagnosis or treatment or failure of medical equipment (Table 4).

**Table 4:** Registries’ reasons for compensation in 36 granted claims following surgical treatment of focal cartilage defects in the knee joint

Reason for granted compensation	N = 36 (%)
Inadequate surgical technique	12 (33.3%)
Hospital-acquired infection	9 (25.0%)
Nerve injury	5 (13.9%)
Delayed diagnosis or treatment	4 (11.1%)
Treatment failure	3 (8.3%)
Spinal headache	2 (5.6%)
Failure of medical equipment	1 (2.8%)

All 8 patients given compensation due to surgical site infection underwent debridement. One patient who underwent debridement was granted compensation due to an infected peripheral vein catheter.

The majority of claims were rejected because good clinical practice was followed or because no causal connection was found. Three claims were rejected because there was no financial loss.

Complaints from public hospitals were compensated more often (31/89) than complaints from private hospitals (5/14) (*p* = 0.004).

A total of €807.086 has been paid in compensation with an average payment of €24.457. In 3 cases the amount of compensation had not yet been settled. The median compensation was €5652, with range €209 - €458.943. The skewed distribution of compensation was caused by one patient, who was granted compensation 10 times higher than the second highest compensation. This patient was a 47-year-old female who sustained a hospital-acquired infection following debridement. This led to almost 2.5 years of sick-leave, explaining the high compensation.

## Discussion

This study highlights the epidemiology of patient compensation claims following articular cartilage surgery in the knee joint over a six years period. The main reasons for compensations were inadequate surgical technique (no further explanation was accessible), hospital-acquired infection, nerve injury and delayed diagnosis or treatment. Most claims filed for compensation due to hospital-acquired infection was granted compensation, all following arthroscopic debridement. Pain was a common reason for patients’ complaint, but is usually not a valid cause of compensation by itself. Our study also finds that women more often file a claim than men [23]. There was no mortality recorded or claims due to wrong-sided surgery.

There was a surprisingly low number of compensation claims identified in Scandinavia in the study period. The true incidence of cartilage procedures is unknown, but the incidence seems to be increasing [24]. Merkely et al. stated that more than 200,000 cartilage procedures were performed annually in America [25], and Engen reported approximately 2500 cartilage procedures are performed annually in Norway [26]. This yields approximately 45,000 cartilage procedures in Scandinavia during the study period. Based on these numbers, one should expect a higher number of compensation claims. We identified 103 compensation claims over a six-year period, an average of 17 complaints annually. This is substantially lower than the findings of Randsborg et al. who identified 24 compensation claims yearly following anterior cruciate ligament reconstruction in Norway alone [27].

We found more compensation claims in Denmark, despite the fact that Sweden has about twice the population size. The reason for this is unclear. We believe it reflects cultural differences, rather than a real difference in the quality of cartilage surgery between the respective countries. In fact, it might indicate that Denmark has a better system of detecting patient injury claims.

Since the introduction of ACI two decades ago [28], this procedure has gained popularity both routinely and in clinical trials, as is the case for mosaicplasty [6, 11, 29]. Nevertheless, compensation claims following mosaicplasty and ACI are almost absent in our material covering three countries for six years. Only two cases of compensation following mosaicplasty or ACI were found. These findings are in line with previous studies stating that major complications following mosaicplasty and ACI are very rare [30–33]. Debridement and MF are low-cost and relatively simple procedures available in smaller hospitals and private clinics that cannot offer the more advanced cartilage procedures, such as mosaicplasty and ACI, which requires highly specialized institutions. The total numbers of debridement and MF performed annually is much higher than mosaicplasty and ACI [26]. This explains the large predominance of complaints by debridement and MF.

Lack of communication and poor patient expectation management are well-known risk factors for compensation claims [34]. It is possible that patients scheduled for mosaicplasty or ACI in highly specialized knee units are better prepared and well informed prior to surgery, and might receive better follow-up, than patients operated in smaller clinics. Furthermore, mosaicplasty and ACI are often considered salvage procedures when simpler interventions have failed. This might alter the patient expectations to these more complex knee surgeries, which again affects the threshold for filing a compensation claim.

Although most complications were related to the surgery, 2 were caused by the anesthesia. This is a reminder that surgery also included risks unrelated to the procedure itself.

Ohrn et al. showed that 23% of all compensation claims to the National Swedish Patient Insurance Company were attributed to orthopedic surgery, whereas Bjerkreim reported that 47% of all compensation claims filed to the NPE were after orthopedic treatment [35, 36]. National health oversights in Scandinavia have reported that patients’ complaints have increased in all three countries in recent years [37]. From 2005, there has been approximately a 10% annual increase in compensation claims.

Although patients have become more aware of the possibility of applying for compensation, our findings indicate that complaints following knee cartilage surgery are fewer than anticipated. The reason for this may be diverse. Perhaps the surgically treated cartilage patients are so troubled by their knee that they have low expectations. Or, although unlikely, the surgery is successful for most of the patients. Another possible reason is the lack of information from health care professionals regarding the opportunity to apply for compensation.

The amount of compensation following arthroscopic surgery varies greatly between countries. In their study of medical malpractice litigation following knee arthroscopy, Shah et al. found an average settlement of $848.331 (€733.486) [17]. We found an average compensation of €24.457. This is almost exactly the same amount of compensation granted following anterior cruciate ligament reconstruction in Norway (€24.200) [27]. This indicates that compensation amount is substantially lower in Scandinavia than in the United States.

The study from the United States by Shah and colleagues reported medical malpractice litigation following arthroscopic surgery [17]. Over 29 years, they reported 162 litigations following knee arthroscopy, yielding less than six litigations annually. This is substantially lower than our findings of 17 compensation claims annually, and they did not specify which treatment was given. Shah. et al. found that 64% of the claims were rejected, similar to our findings. They reported musculoskeletal complaint (listed as chronic pain, stiffness and unsatisfactory result), infection and deep vein thrombosis as the three main reasons for compensation claims. Different from our finding, Shah reported 19 deaths and 10 cases of wrong-sided surgery, whereas we registered no deaths or wrong-sided surgery. Our study differs from theirs as we only report compensation claims following treatment of FCDs and have excluded other common arthroscopic procedures such as ligament reconstruction and meniscal procedures. On this basis, our findings supplement the results of Shah et al. and add further knowledge in compensation claims following arthroscopic surgery and FCDs in particular.

The Scandinavian countries use the no-blame principle for practitioners in handling compensation claims, eliminating the fault criterion. This implies that no data is shared with the regulatory authorities, and cases are usually handled outside the legal system where the insurance provider recovers the cost of a claim from the liable party. The no-fault approach system is not unique in Scandinavia, as this is found in Finland, France, New Zealand and two American jurisdictions (Florida and Virginia) [38, 39]. The opposite of a non-fault claim is the court-based tort law system, where the liable party is responsible for the cost of a claim based on the fault criterion. This system is among other countries used in the United Kingdom and most American jurisdictions, where patient injury compensation claims are handled by the juridical system [12, 23]. Both these systems have their pros and cons, but one major advantage of the no-fault system is that it generates novel patient safety data for research and learning [40].

The most obvious and major limitation to this study is that we do not know the absolute numbers of each procedure performed in Scandinavia during the study period. Therefore, we cannot estimate the risk of compensation for the various surgical techniques. However, our study demonstrates the epidemiology of compensation claims and highlights the need of national cartilage registries. The study population was based on a set of predefined diagnosis and surgical procedures. Any kind of mislabeling of these by the orthopedic surgeon may cause some patients not to be included, introducing an inclusion bias. By using a broad range of diagnosis and surgical procedures and not only cartilage specific codes, we have tried to reduce this error. The total number of study subjects are relatively low, and may affect the results of this study.

The Scandinavian registries do not comprise all complications encountered after cartilage surgery. Some patients might have suffered a complication that would have led to compensation, but never filed a complaint to the registries. These factors may contribute to different biases to the cases available in the databases. The demographics do not include information such as ethnicity, socioeconomic status and insurance status, factors that we would like to illuminate.

Patient expectation management is important following cartilage restoration surgery. Our study is the first national report on compensation claims after cartilage injury and has focused on compensation claims after surgical treatment of focal cartilage defects in the knee. Knowledge of compensation claims following conservative treatment is lacking and should be highlighted in the future in the work on patient safety. Our study has demonstrated that the claim rate is low following these injuries and should be assessed in future research by validating patient’s compensation claims by comparing institutional data with the filed compensation claims. Little is known whether health care professionals fail to inform patients of the possibility to file a compensation claim following a treatment injury. This topic should be addressed in future research.

## Conclusions

Compensation claims following cartilage surgery in the knee are rare, and may suggest a lack of patient information on compensation claims from health care professionals. Establishing nationwide cartilage registries can add further knowledge on this troublesome disease.

## Data Availability

This study brought together existing data obtained upon request and subject to license restrictions from the National Swedish Patient Insurance Company, the Danish Patient Insurance Association and the Norwegian System of Patient Injury Compensation. The authors declare that the data supporting the findings of this study are available within the article.

## Abbreviations

ACI: Autologous chondrocyte implantation
FCDs: Focal cartilage defects
ICD-10: International Statistical Classification of Diseases and Related Health Problems 10th Revision
MF: Microfracture
NCSP: NOMESCO Classification of Surgical Procedures
NPE: Norwegian System of Patient Injury Compensation
